# A panel of 32 AIMs suitable for population stratification correction and global ancestry estimation in Mexican mestizos

**DOI:** 10.1186/s12863-018-0707-7

**Published:** 2019-01-08

**Authors:** Alicia Huerta-Chagoya, Hortensia Moreno-Macías, Juan Carlos Fernández-López, María Luisa Ordóñez-Sánchez, Rosario Rodríguez-Guillén, Alejandra Contreras, Alfredo Hidalgo-Miranda, Luis Alberto Alfaro-Ruíz, Edgar Pavel Salazar-Fernandez, Andrés Moreno-Estrada, Carlos Alberto Aguilar-Salinas, Teresa Tusié-Luna

**Affiliations:** 1 CONACYT, Instituto Nacional de Ciencias Médicas y Nutrición Salvador Zubirán, Ciudad de Mexico, Mexico; 2 Departamento de Economía, Universidad Autónoma Metropolitana, Ciudad de Mexico, Mexico; 3 Departamento de Genómica Computacional, Instituto Nacional de Medicina Genómica, Ciudad de Mexico, Mexico; 4 Unidad de Biología Molecular y Medicina Genómica, Instituto Nacional de Ciencias Médicas y Nutrición Salvador Zubirán, Ciudad de Mexico, Mexico; 5 Instituto Nacional de Medicina Genómica, Ciudad de Mexico, Mexico; 6 Fox Chase Cancer Center, Philadelphia, USA; 7 Laboratorio de Genómica del Cáncer, Instituto Nacional de Medicina Genómica, Ciudad de Mexico, Mexico; 8 Laboratorio Nacional de Genómica para la Biodiversidad (LANGEBIO-UGA), CINVESTAV, Iraputato, Guanajuato, Mexico; 9 Departamento de Endocrinología y Metabolismo, Instituto Nacional de Ciencias Médicas y Nutrición Salvador Zubirán, Ciudad de Mexico, Mexico; 10 Departamento de Medicina Genómica y Toxicología Ambiental, Instituto de Investigaciones Biomédicas, UNAM, Ciudad de Mexico, Mexico

**Keywords:** AIM, Ancestry, Population stratification, Association study, Mexican mestizos

## Abstract

**Background:**

Association studies are useful to unravel the genetic basis of common human diseases. However, the presence of undetected population structure can lead to both false positive results and failures to detect genuine associations. Even when most of the approaches to deal with population stratification require genome-wide data, the use of a well-selected panel of ancestry informative markers (AIMs) may appropriately correct for population stratification. Few panels of AIMs have been developed for Latino populations and most contain a high number of markers (> 100 AIMs). For some association studies such as candidate gene approaches, it may be unfeasible to genotype a numerous set of markers to avoid false positive results. In such cases, methods that use fewer AIMs may be appropriate.

**Results:**

We validated an accurate and cost-effective panel of AIMs, for use in population stratification correction of association studies and global ancestry estimation in Mexicans, as well as in populations having large proportions of both European and Native American ancestries.

Based on genome-wide data from 1953 Mexican individuals, we performed a PCA and SNP weights were calculated to select subsets of unlinked AIMs within percentiles 0.10 and 0.90, ensuring that all chromosomes were represented. Correlations between PC1 calculated using genome-wide data versus each subset of AIMs (16, 32, 48 and 64) were *r*^*2*^ = 0.923, 0.959, 0.972 and 0.978, respectively. When evaluating PCs performance as population stratification adjustment covariates, no correlation was found between *P* values obtained from uncorrected and genome-wide corrected association analyses (*r*^*2*^ = 0.141), highlighting that population stratification correction is compulsory for association analyses in admixed populations. In contrast, high correlations were found when adjusting for both PC1 and PC2 for either subset of AIMs (*r*^*2*^ > 0.900). After multiple validations, including an independent sample, we selected a minimal panel of 32 AIMs, which are highly informative of the major ancestral components of Mexican mestizos, namely European and Native American ancestries. Finally, the correlation between the global ancestry proportions calculated using genome-wide data and our panel of 32 AIMs was *r*^*2*^ = 0.972.

**Conclusions:**

Our panel of 32 AIMs accurately estimated global ancestry and corrected for population stratification in association studies in Mexican individuals.

**Electronic supplementary material:**

The online version of this article (10.1186/s12863-018-0707-7) contains supplementary material, which is available to authorized users.

## Background

The goal of genetic association studies is to identify DNA genetic variants that vary systematically between individuals with different disease states (e.g. cases versus controls) and could therefore represent the effects of risk-enhancing or protective alleles [1]. These studies have been useful to unravel the genetic basis of common human diseases. However, a well-known constrain of association studies is the presence of undetected population structure, which can lead to both false positive results and failures to detect genuine associations. These concerns have influenced the design and interpretation of association studies. Even when levels of population structure in many ethnic groups are typically small, the problem is critical if association studies are performed in recently admixed populations, such as Mexicans [2].

Population stratification refers to the fact that the study population consists of subpopulations with heterogeneous genetic background. Disease prevalence and allele frequencies may be different among groups due to the stratification rather than a biological mechanism. In this case, it is said that the stratification confounds the association between genetic variants and phenotype. If disease prevalence also differs across the subpopulations, then the proportions of cases and controls sampled from each subpopulation will tend to differ, as well as the allele frequencies between cases and controls at any *locus* at which the subpopulation differ. For example, if the cases of population A disproportionately represent a genetic subgroup, then any genetic variant with higher allele proportions than the control group will be falsely associated with the case status [3].

However, if the population strata are properly identified they can be adjusted for in the analysis. Most of the approaches to deal with population stratification require genome-wide data, such as the genomic control, inference of global genetic ancestry and the mixed-models [4]. However, for some association studies (e.g. candidate gene approaches), it may be unrealistic to genotype the many markers necessary to estimate ethnicity thus, avoiding false positive results. In such cases, methods that use fewer markers, specifically ancestry informative markers (AIM), may be appropriate and more cost-effective [2]. An AIM is a marker whose allele proportions differ between the ancestral populations that contributed to an admixed population. Specifically, the Mexican population was mainly originated from the admixture of European and Native American ancestral populations, with a small African ancestry contribution ranging from 1.92 to 6.91% across the country [5]. Even when genome-wide data is not available, a Principal Component Analysis (PCA) using a well-selected panel of AIMs can potentially correct for population stratification [6]. To date, few panels of AIMs have been developed for Latino populations, in part, due to the lack of genome-wide information from Mestizo and Native American populations. Moreover, they are usually large sets of AIMs comprising more than 100 markers.

## Methods

The aim of this study was to design an accurate and cost-effective panel of AIMs for population stratification correction in association analyses in Mexicans. We used genome-wide data as the gold standard for assessing the performance of our proposed panel.

### Selection of AIMs

A total of 2067 Mexican individuals recruited at the Instituto Nacional de Ciencias Médicas y Nutrición Salvador Zubirán from the SIGMA T2D Consortium [7] were genotyped using Illumina Human Omni 2.5 SNP array. Only biallelic SNP variants were considered. Genotyping quality control excluded SNPs and samples with 2% or more missing data, as well as SNPs that had < 1% minor allele frequency. We also removed related individuals. After quality control, 1953 Mexican individuals and 1.4 M of common SNPs were used for analysis. We were interested in selecting common variation in the Mexican population that potentially had large differences between the two main ancestral populations, the European and the Native American ancestries (42% vs. 55%, respectively) [8]. PCA was performed on quality-controlled genome-wide SNPs using EIGENSOFT [9].

SNP weights from the PCA were calculated, sorted on the absolute value and used to select four nested subsets of AIMs (16, 32, 48 and 64) with the highest SNP weight scores. To compute the SNP weights, EIGENSOFT first normalizes the genotypes and performs PCA on normalized genotypes. Then, it computes SNP weights using PCs, corresponding to eigenvalues and normalized genotypes [10]. Since PC1 showed a primary role for discriminating between the two main parental ancestries of the Mexican population (the European and the Native American ancestries), it was the only PC considered for AIMs selection (Additional file 1: Figure S1).

For the selection of AIMs, we used the top 20 SNPs weights per chromosome. In order to determine the number of included AIMs per chromosome, we considered the Denver system of classification of chromosomes. A higher number of SNPs were included for large (groups A and B) than for medium (groups C, D and E) or small chromosomes (groups F and G). For large chromosomes, more than 1 AIM was selected. It was sought that all chromosomes were represented. In addition, all the SNPs required not to be in linkage disequilibrium (*r*^*2*^ < 1 × 10^− 8^).

The number of AIMs contained in each panel was decided based on the fact that an additional objective of the study was to validate the subset of selected AIMs in a commercial genotyping platform (QuantStudio 12 K Flex Real Time PCR System Open Array). For this sake, nested subsets were formed using multiples of 16 AIMs. We chose an arbitrary limit number of AIMs in order to get a minimum panel with smaller density as compared with the previously published ones (ranging from 100 to 446 AIMs) [11–13], as well as on cost-efficient considerations.

### Ancestry index computation

In order to mimic what is done when genome-wide data is available, PCAs were computed for each subset of AIMs using EIGENSOFT [9], following the same methodology explained above.

### Selection of one subset of AIMs

The SIGMA T2D Consortium is a project aimed at characterizing the genetic basis of type 2 diabetes (T2D), in Mexican and other Latino populations [14]. We used the results of this genome-wide association study to assess the performance of our proposed subsets of AIMs.

The association of SNP genotype with T2D was evaluated through logistic regression models adjusted for gender, age and body mass index. In addition, the top two principal components (PC1 and PC2) were included as adjustment covariates for population stratification as they capture most of the ancestry variance. Models without PCs adjustment (uncorrected for population stratification) were also performed. Genomic inflation factor (λ, lambda) was calculated and quantile-quantile (QQ) plots were created using *P* values unadjusted for lambda.

Based on the above, the performance of the proposed subsets of AIMs was assessed using two criteria: 1) the correlation between genome-wide PCs (gold-standard) and those obtained from each subset of AIMs and 2) the correlation between the *P* values obtained in the association analysis adjusted for genome-wide PCs (gold-standard) and those *P* values obtained when the AIMs subsets’ PCs were used instead. Additional correlations included the gold-standard versus uncorrected models.

Aiming to a candidate gene approach, we specifically compared the obtained association *P* values of four previously published T2D risk SNPs: rs13342232 (*SLC16A11*), a private Native American ancestral variant [7], as well as rs7903146 (*TCF7L2*), rs2237897 (*KCNQ1*) and rs7754840 (*CDKAL1*), which have been replicated in different ethnic populations [15].

Thus, we selected the subset with the minimal number of AIMs but with an optimal performance for population stratification correction, as demonstrated by high correlations for both PC1 and association *P* values with our gold standard (genome-wide data). The resulting minimal subset of AIMs was further assessed by comparing its performance against three previously published panels of Latino populations [11–13] (Additional file 2: Table S1). When available, genotypes from each panel were extracted from the 1953 Mexican sample; otherwise imputed data (info score = 1) was used. SNP imputation was performed by pre-phasing with HAPI-UR version 1.01 [16] and imputation with IMPUTE version 2.2.0 [17]. We used the 1000 Genomes Phase I integrated variant set (build 37 and haplotype release date in August, 2012) as our reference panel. In no more than 10 AIMs per panel, a tag SNP (r^2^ > 0.9) was used, as no genotype nor imputed information was available. Comparisons were performed as described above.

### Validation of the selected subset of AIMs

Once we selected the most accurate and cost-effective panel, we performed a validation step, in order to: 1) assess if the sample size was a determinant factor in the accuracy of the population stratification correction, 2) evaluate if the accuracy of the population stratification correction of the AIMs panels was preserved, in spite of the known fluctuations in the ancestral proportions throughout Mexico, 3) validate its accuracy in correcting for population stratification in association analyses of a non-metabolic trait in an independent sample and 4) assess its performance for global ancestry estimation.

For the first purpose, we randomly generated three smaller samples (*N* = 1500, 1000 and 500). PCs were recomputed for each subsample, as well as the correlation between our gold standard PC1 versus the PC1 obtained through our selected subset of AIMs.

In order to assess the accuracy of the population stratification correction through the Mexican country, we stratified the sample according to the state of birth of each participant. We considered four representative regions of Mexico as described in the National Health and Nutrition Survey, 2012. States grouped in each region share geographical, as well as socioeconomic characteristics [18]. Global ancestry of the individuals born in each region of Mexico was calculated using genome-wide data and parental information from the 1000 Genomes Project [19] with ADMIXTURE [20] at K = 3. Correlation between the gold standard PC1 and PC1 calculated using the panels for each region of Mexico was evaluated.

In favor of assessing the accuracy in correcting for population stratification in association analyses of non-metabolic traits, we used height as the outcome in an independent Mexican sample with available genome-wide genetic data [SIGMA phase 2]. Again, the first top PCs and *P* values were compared with its respective gold standard. Specifically, we compared the obtained association *P* values of two previously published height related SNPs: rs143384 (*GDF5*) and rs1536147 (*ERGIC3*) [21].

**Fig. 1:**
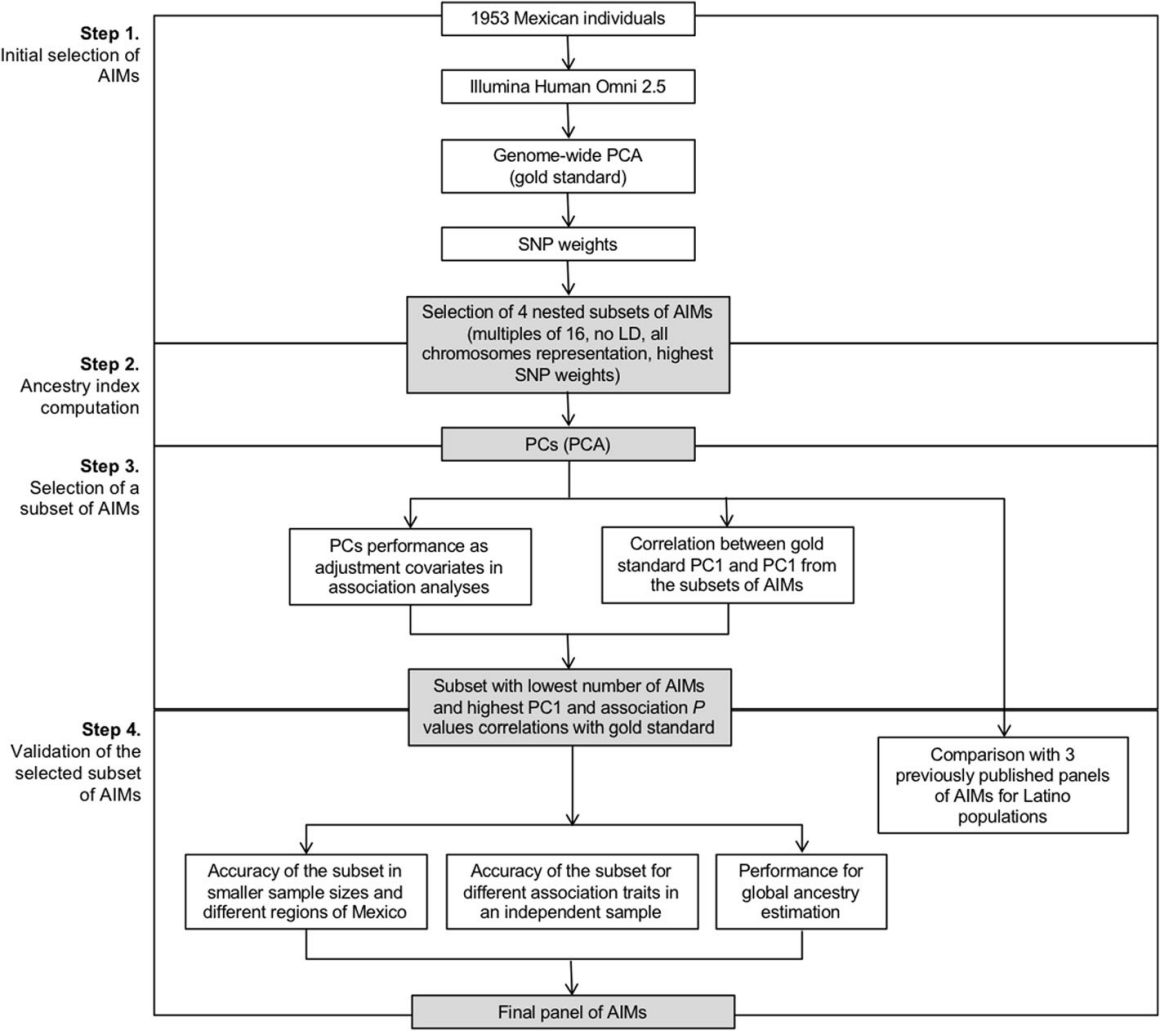
AIMs selection algorithm. *Step 1* included the initial selection of 4 nested subsets of AIMs based on SNP weights of a PCA using 1.4 M of SNPs of 1953 Mexican individuals. *Step 2* comprised the ancestry index computation for each nested subset of AIMs in order to evaluate its performance for population stratification. *Step 3* involved the selection of the most accurate subset with the minimal number of AIMs, as well as comparing with three previously published panels. *Step 4* comprised a validation to assess the accuracy of the minimal panel for population stratification correction in small samples and different regions of Mexico, as well as in a non-metabolic trait association study and global ancestry estimation

Regarding to the global ancestry estimation, we performed a comparative analysis using data from the 1000 Genomes Project [19] and the Mexican Genome Diversity Project [5, 8]. We selected 95 non-related individuals from the European Utah population (CEU); plus 38 Mexican Native individuals (Maya and Zapoteca Native groups). After thinning the original dataset down to 1.4 million autosomal bi-allelic sites and applying a ≥ 1% MAF filter with PLINK [22]. we obtained a final file containing 336,298 SNPs. This dataset was analyzed with ADMIXTURE at K = 2 and the results saved as reference. Then, we extracted only the 32 SNPs used as AIMs in this study from the same samples and ran ADMIXTURE again at K = 2. After merging the results from both ADMIXTURE runs, we calculated the differences between the runs. A diagram of the methodology is shown in Fig. 1.

## Results

**Fig. 2:**
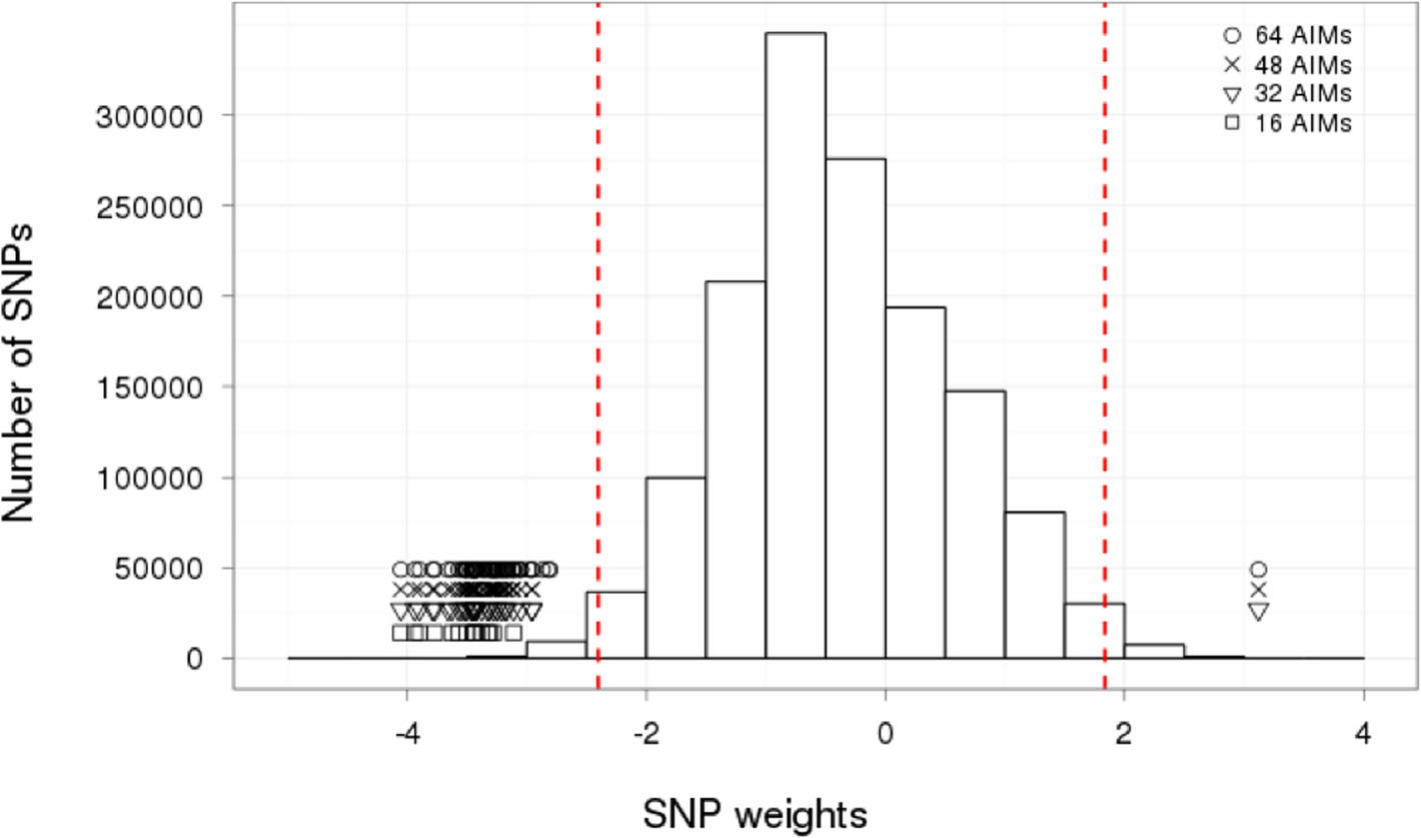
SNP weights distribution. Histogram of the SNP weights computed from a PCA using 1.4 M of SNPs in a 1953 Mexican sample. Points represent each of the four nested subsets of AIMs assessed. Red dashed lines show percentiles 0.01 and 0.99

**Fig. 3:**
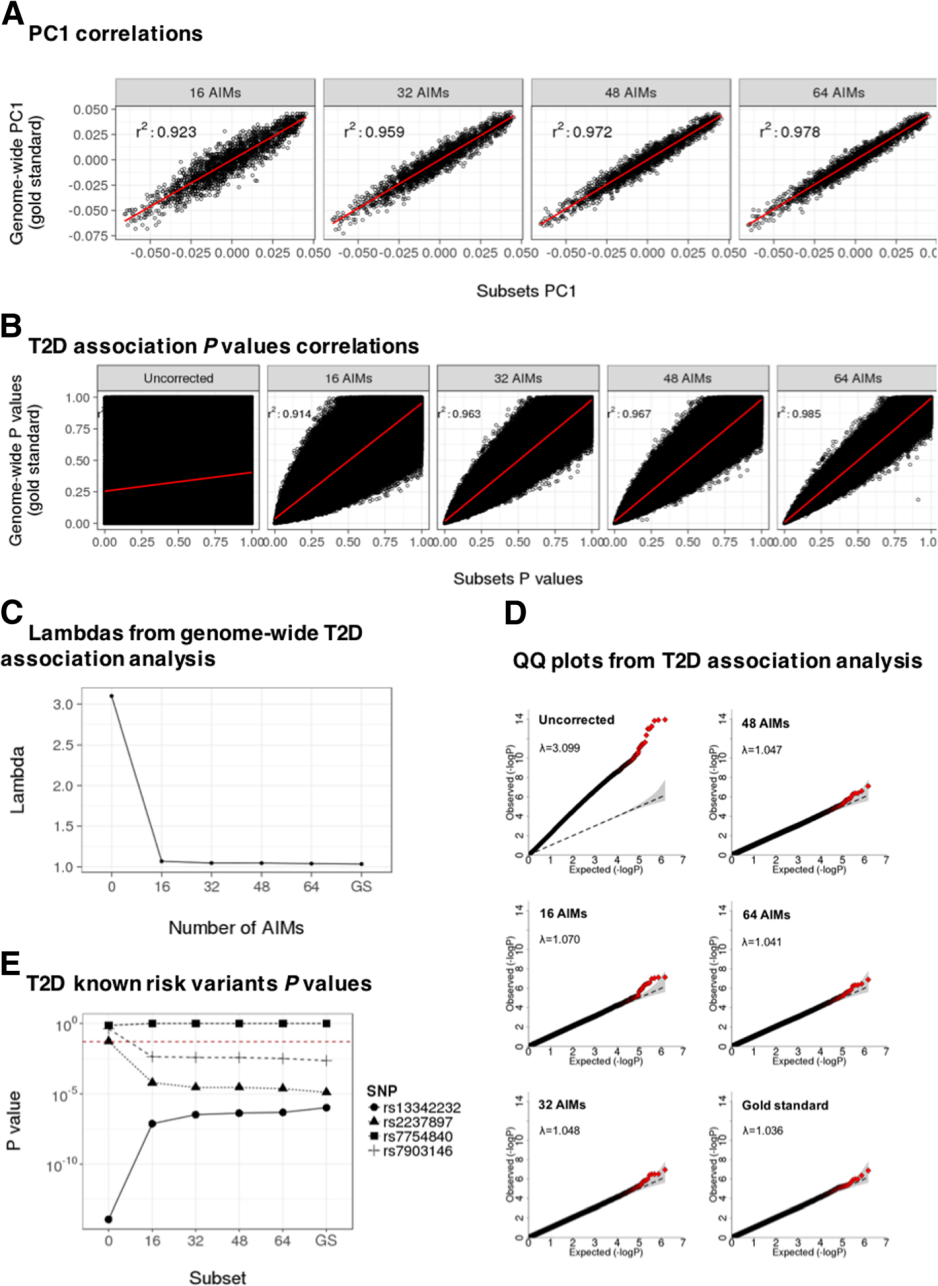
Selection of an accurate subset of AIMs. **a**. Correlation between gold standard PC1 and PC1 calculated using four nested subsets of AIMs. **b**. Correlation between gold standard T2D association *P* values and those obtained from T2D association analyses uncorrected for population stratification, as well as corrected for PC1 and PC2 calculated using four nested subsets of AIMs. **c**. Inflation factors (lambdas) calculated using gold standard T2D association *P* values, uncorrected *P* values for population stratification, as well as *P* values corrected for PC1 and PC2 calculated using four nested subsets of AIMs. **d**. QQ plots of *P* values from population stratification uncorrected and corrected T2D association analyses. **e**. *P* values obtained from previously well-known T2D risk variants when association analyses were uncorrected or corrected for population stratification using PC1 and PC2 calculated from either gold standard or four subsets of AIMs. GS: Gold Standard

After genome-wide PCA, 28,770 out of the 1.4 million genetic variants showed a SNP weight value within percentiles 0.01 and 0.99 (− 2.404, 1.835, respectively), which were used for AIMs selection (Fig. 2). Correlations between PC1 calculated using genome-wide data (gold standard) versus each of the four subsets of AIMs were *r*^*2*^ = 0.923, 0.959, 0.972 and 0.978 (subsets of 16, 32, 48 and 64 AIMs, respectively) (Fig. 3a). When evaluating PCs performance as population stratification adjustment covariates in association analyses, no correlation was found between *P* values obtained from uncorrected and genome-wide corrected association analyses (*r*^*2*^ = 0.141), highlighting that population stratification correction is compulsory for association analysis in admixed populations. In contrast, high correlations were found when adjusting for both PC1 and PC2 for either subset of AIMs (*r*^*2*^ > 0.900) (Fig. 3b). The inflation factor diminished from λ = 3.10 for uncorrected analysis to λ < 1.07 for either subset of AIMs adjusted analysis. Remarkably, inflation factor for genome-wide corrected analysis was λ =1.036, a value that does not depart considerably from the lambdas obtained by including PCs calculated using the four subsets of AIMs (Fig. 3c). Correction of the population stratification genomic inflation, by the inclusion of PC1 and PC2 as covariates was also demonstrated by QQ plots (Fig. 3d).

When assessing the association of four previously known T2D-risk SNPs, we found that uncorrected analyses either over, equal or underestimated the association *P* values. In the case of over or underestimation, correction for PC1 and PC2 restored the *P* values to those obtained from association analyses adjusted with PCs calculated using genome-wide data (Fig. 3e).

### The subset of 32 AIMs was the minimal but accurate and cost-effective panel

Even though the four subsets of AIMs adequately corrected for population stratification, the subset of 32 AIMs was selected based on the following: 1) correlation between *P* values obtained from the 32 AIMs subset did not depart considerably from *P* values obtained from 48 and 64 AIMs subsets, 2) inclusion of PC1 and PC2 in logistic regression models restored the inflation caused by population stratification to genome-wide levels, as demonstrated by lambdas values and calibrated QQ plots (λ = 3.10, 1.048 and 1.036 for uncorrected, our 32 AIMs and gold standard corrections, respectively) and 3) our subset of 32 AIMs approximates genome-wide coverage by including 1 to 2 SNPs in all chromosomes, with the exception of chromosome 18 where the SNPs did not show proper SNP weight values.

### Our panel of 32 AIMs performs well as compared with more extensive previously published panels

**Fig. 4:**
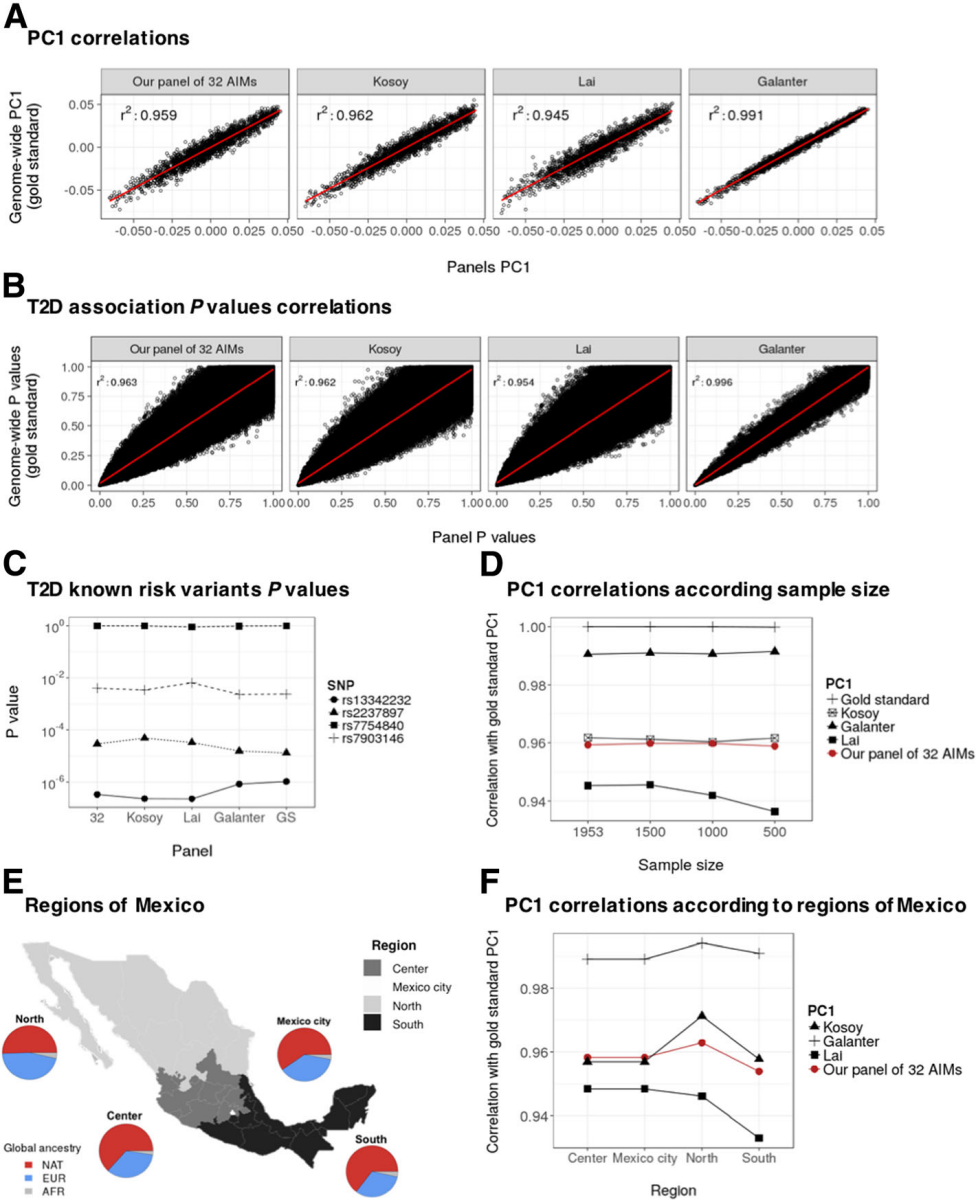
Validation of our proposed panel of 32 AIMs. **a**. Correlations between gold standard PC1 and PC1 calculated using our subset of 32 AIMs and three previously published panels of AIMs. **b**. Correlation between *P* values obtained from association analysis corrected for gold standard PC1 and PC2 and *P* values obtained from association analyses corrected for PC1 and PC2 calculated using our subset of 32 AIMs, as well as from association analysis corrected for PC1 and PC2 using three previously published panels of AIMs. **c**. *P* values obtained from previously well-known T2D risk variants when association analyses were corrected for population stratification using gold standard PC1 and PC2, as well as PC1 and PC2 calculated from either our subset of 32 AIMs or three previously published panels of AIMs. **d**. Correlations between gold standard PC1 and PC1 calculated using our subset of 32 AIMs and three previously published panels of AIMs using several sample sizes. **e**. Map of the four geographic regions of Mexico and the ancestry proportions for each one. **f**. Correlations between gold standard PC1 and PC1 calculated using our subset of 32 AIMs and three previously published panels of AIMs using the four geographic regions of Mexico. GS: Gold Standard

Once designed, we compared the performance of our 32 AIMs panel with three previously published panels [11–13]. All of them comprise 100 or more AIMs. A summary of such published panels is available in Additional file 2: Table S1. High correlation was found between gold standard PC1 and PC1 calculated using our subset of 32 AIMs and the three previously published panels of AIMs (*r*^*2*^ > 0.90) (Fig. 4a). The same was observed when comparing the *P* values obtained from genome-wide corrected association analysis and *P* values obtained from the association analysis corrected for PC1 and PC2 calculated using either our subset of 32 AIMs or the three previously published panels of AIMs (*r*^*2*^ > 0.96) (Fig. 4b). In addition, T2D association *P* values of the four previously known T2D risk SNPs were essentially the same when analyses were corrected using PC1 and PC2 obtained using either genome-wide data, our panel of 32 AIMs or any of the published panels of AIMs (Fig. 4c).

### Our panel of 32 AIMs performs well when using small samples

Regarding the accuracy of the population stratification correction when including small sample sizes, we found that the correlation between the gold standard PC1 and the PC1 calculated using our final subset of 32 AIMs was high (*r*^*2*^ > 0.95), even when randomly reducing the sample size to less than half (500 individuals) (Fig. 4d).

### Our panel of 32 AIMs is equally accurate for the distinct regions of Mexico

According to previous reports [5], we found that the individuals born in the northern region of Mexico showed a lower Native American ancestry proportions, as compared to the individuals born in the rest of the country. In this study, the Native American ancestry proportions of the four geographic regions of Mexico were: North region 50.5%, Center region 63.3%, Mexico City region 59.6% and South region 64.6% (Fig. 4e). In spite of the above, the correlation between the PC1 calculated using genome-wide data and the PC1 calculated using our final subset of 32 AIMs was high (*r*^*2*^ > 0.95), even when separately analyzing the individuals born in each of the four different regions of Mexico (Fig. 4f).

When comparing with the three previously published panels, similar correlations were found even when our final subset comprises a lesser number of AIMs (32 AIMs) (Fig. 4d and f).

### Our panel of 32 AIMs is accurate when analyzing a non-metabolic phenotype and estimating global ancestry

**Fig. 5:**
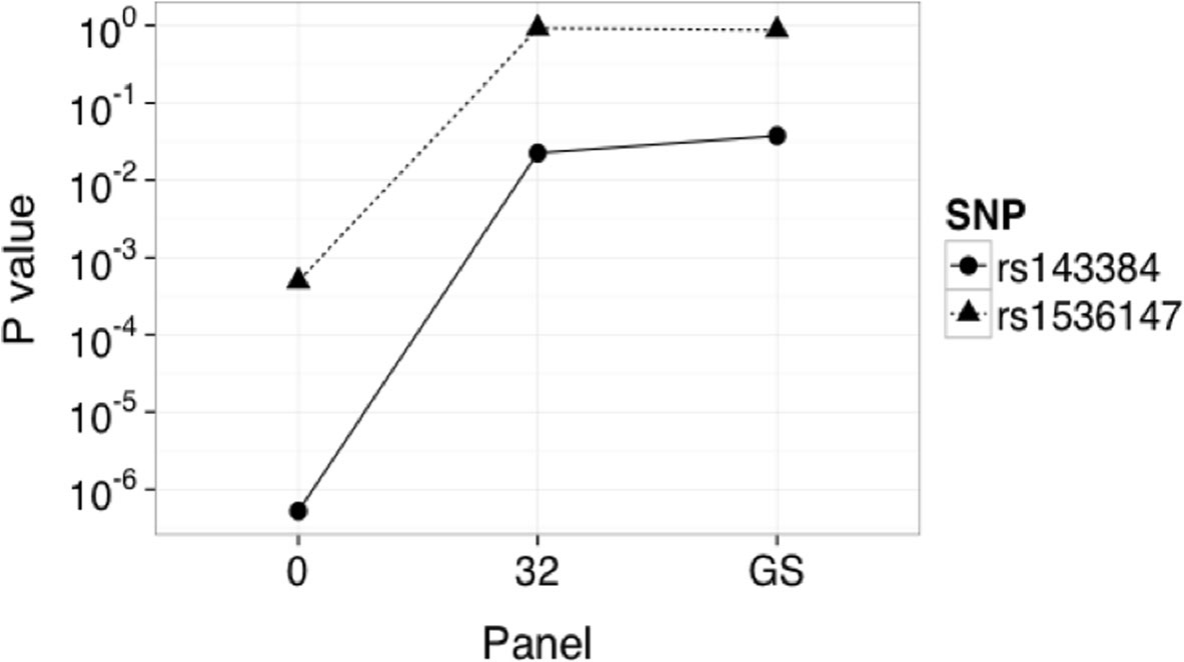
Accuracy of our panel of 32 AIMs in a non-metabolic trait association study. *P* values obtained from previously well-known height related variants when association analyses were uncorrected or corrected for population stratification using gold standard PC1 and PC2 or those calculated using our panel of 32 AIMs. GS: Gold Standard

When validating our panel of 32 AIMs for non-metabolic association analyses, we found that the association for height trait was strongly influenced by ancestry fluctuations. Inclusion of PC1 and PC2 adequately corrected the association analyses for population stratification (rs143384: uncorrected β = 0.026, *P* = 5.23 × 10^− 07^; our 32 AIMs β = 0.011, *P* = 0.020; gold standard β = 0.010, *P* = 0.038 and rs1536147: uncorrected β = 0.020, *P* = 0.0005; our 32 AIMs β = − 0.00001, *P* = 0.998; gold standard β = − 0.0008, *P* = 0.871) (Fig. 5).

**Fig. 6:**
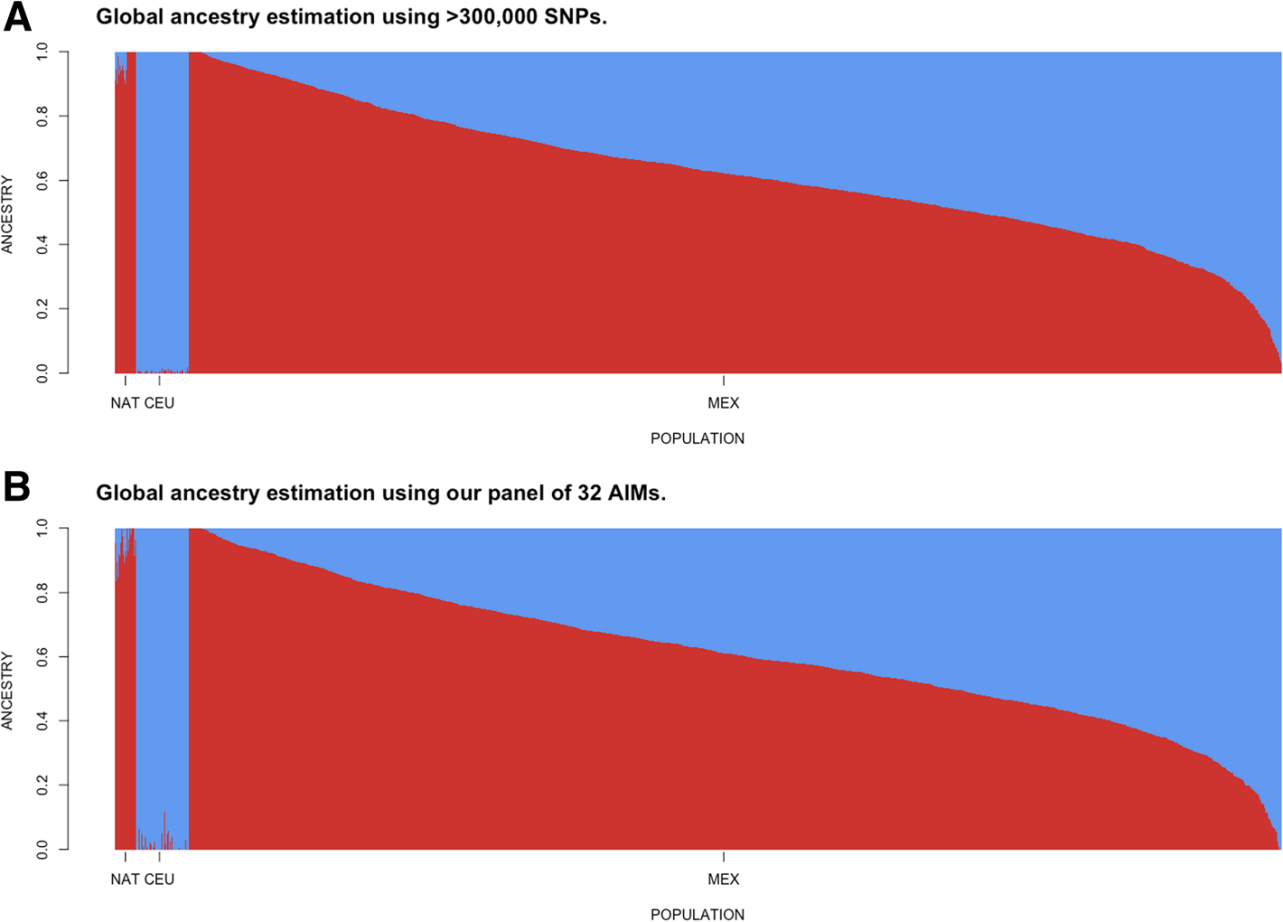
Performance of our panel of 32 AIMs for global ancestry estimation. Global ancestry estimation for the two main ancestral populations of Mexican people using **a** > 300,000 random SNPs and **b** our panel of 32 AIMs

Moreover, our panel of 32 AIMs demonstrated a good performance for global ancestry estimation. The correlation between the global ancestry proportions calculated using genome-wide data and our panel of 32 AIMs was *r*^*2*^ = 0.972 (Fig. 6).

Finally, the absolute value of allele frequency difference (delta) between parental populations for each of the 32 AIMs was > 0.77. As expected, mestizo individuals included in this study showed intermediate allele frequencies (Additional file 3: Table S2).

## Discussion

In this study, we identified and validated a minimum viable panel of 32 AIMs, which accurately corrects for population stratification in association studies in Mexicans. In addition, it accurately estimates the global ancestry proportions of the two major parental populations of Mexican mestizos –the European and Native American ancestries–. For this purpose, we used previously generated genome-wide genotyping data a Mexican cohort (~ 1.4 M of SNPs after QC). Given that the first principal component showed a relevant role for discriminating between European and Native American ancestry, we used the SNP weights from PCA as a selection criteria of the most meaningful markers of ancestry. Accordingly, the selected AIMs showed an allele frequency difference between European and Native American individuals > 0.77.

Our results confirmed that population stratification correction is a compulsory step when performing association analyses in admixed populations, such as Mexican mestizos, particularly, when studying diseases whose incidence is affected by ancestry. Avoiding correction not only overestimated the association *P* values, but also led to oversight of real associations. Even though our panel was selected from a case-control sample design for a metabolic trait, it is also appropriate for correction of non-metabolic traits association studies, as well as for non-case-control approaches (i.e. continuous traits). It is important to emphasize that the use of our panel was intended for population stratification correction in association analyses, but its use for other purposes, such as global ancestry estimation, will also result in accurate computations. An example of the further use of such global ancestry estimations includes the supervised sample selection when, for example, exome sequencing could result in the discovery of new genetic variants in individuals with higher Native American ancestry.

Although the costs for genome-wide genotyping are decreasing, the use of a limited panel of AIMs for ancestry estimation is useful, particularly if resources are limited. Our panel is cost-effective when analyzing few markers instead of genome-wide data (e.g. candidate gene approaches). Moreover, its use is not restricted to the correction of the association of SNP genotypes with clinical traits, but also to the correction of eQTLs. It would be useful in various clinical assays, when evaluating for example, drug effectiveness in Mestizo populations, as a method to efficiently correct for confounding variables in case-control studies. Despite its low number of AIMs and given that the PCs are individually computed, our panel is accurate when including small sample sizes (i.e. hundreds of individuals) and individuals from different geographic regions of Mexico. Even more, its use may be extended to non-Mexican populations as long as their mixture is mainly the result of Native American and European global ancestries. Nonetheless, because the panel was not designed to identify African ancestry, its use for populations with high African ancestry (> 10%) should be pursued with caution. Based on the above and in order to favor its use among research groups and potentially increase data sharing, we standardized its genotyping in an easy and cost-efficient array. Although most of the 32 AIMs are included in any of the commercial genotyping arrays available (Additional file 4: Table S3), this panel is available using the QuantStudio 12X Flex Real Time PCR System Open Array.

For those users with no experience in command line, required for EIGENSTRAT computations, we share an R script (Additional file 5: Additional_Compute_PCs.R). PC computations obtained either with EIGENSTRAT or R script are equal. In order to better represent the ancestral founder populations of Mexican mestizos, we also share the genotypes of the 32 AIMs from 38 Native individuals (non-related Zapoteca and Maya from the MGDP Project [5, 8]) (Additional file 6: Additional_Parentals_AIMs.txt). To facilitate the computations, the file also includes public genotypes from 95 European individuals (non-related CEU from 1000G Project [19]).

## Conclusions

We identified and validated the most minimum panel of AIMs, to date. It is composed of 32 AIMs and accurately corrects for population stratification in association studies in Mexicans, as well as estimates the global ancestry proportions of the major ancestral components of Mexican mestizos, namely European and Native American ancestries.

## Additional files

Additional file 1: **Figure S1.** Mexican mestizos projection over parental populations using our panel of 32 AIMs. Red points represent Native American individuals (NAT), blue points represent European individuals (CEU) and green points represent Mexican Mestizos (MEX). (DOCX 168 kb)

Additional file 2: **Table S1.** Summary of the three previously published panels of AIMs used for comparisons in this study. (DOCX 12 kb)

Additional file 3: **Table S2.** Panel of 32 AIMs proposed in this study and allele frequencies. (DOCX 16 kb)

Additional file 4: **Table S3.** Commercial genotyping arrays where our subset of 32 AIMs is available. (DOCX 13 kb)

Additional file 5: **Additional_Compute_PCs.R.** R script for PCs computation using genotypes of our subset of 32 AIMs. (R 3 kb)

Additional file 6 **Additional_Parental_AIMs.txt.** Genotypes of our subset of 32 AIMs from the two parental populations of Mexican mestizos. (TXT 10 kb)

## Abbreviations

AIM: Ancestry informative marker
CEU: European Utah population
PCA: Principal component analysis
QQ: Quantile-quantile
T2D: Type 2 diabetes
