# Supplementary material for: A panel of 32 AIMs suitable for population stratification correction and global ancestry estimation in Mexican mestizos
Source: BMC Genet. 2019 Jan 8;20:5. doi: 10.1186/s12863-018-0707-7 (PMC6323778; doi:10.1186/s12863-018-0707-7)
Supplement: Supplementary file 1 — Figure S1. Mexican mestizos projection over parental populations using our panel of 32 AIMs. Red points represent Native American individuals (NAT), blue points represent European individuals (CEU) and green points represent Mexican Mestizos (MEX). (DOCX 168 kb) [file 12863_2018_707_MOESM1_ESM.docx]

|  |
| --- |
| **Additional Figure 1. Mexican mestizos projection over parental populations using our panel of 32 AIMs.** Red points represent Native American individuals (NAT), blue points represent European individuals (CEU) and green points represent Mexican Mestizos (MEX). |
