# Supplementary material for: A panel of 32 AIMs suitable for population stratification correction and global ancestry estimation in Mexican mestizos
Source: BMC Genet. 2019 Jan 8;20:5. doi: 10.1186/s12863-018-0707-7 (PMC6323778; doi:10.1186/s12863-018-0707-7)
Supplement: Supplementary file 2 — Table S1. Summary of the three previously published panels of AIMs used for comparisons in this study. (DOCX 12 kb) [file 12863_2018_707_MOESM2_ESM.docx]

| **Additional Table 1. Summary of the three previously published panels of AIMs used for comparisons in this study.** | | | | |
| --- | --- | --- | --- | --- |
| **Reference** | **Number of AIMs** | **Type of AIMs** | **Characteristics of the AIMs** | **Parental populations** |
| Kosoy, *et al.*, 2009 | 128 | SNPs | Informative for k=4 populations (European, Native American, African, Asian) | **European:** 60 CEU, 128 European Americans  **Native American:** 29 Nahuas, 40 Mexican Americans, 26 Mexicans, 28 Puerto Ricans  **African:** 56 Yorubas, 19 Bini West Africans  **Asian:** 43 Chinese, 43 Chinese American, 43 Japanese, 8 Vietnamese American, 1 Koreano American, 45 Filipino American, 2 East Asian American, 3 Japanese American, 64 South Asian Indian Americans |
| Lai, *et al.*, 2009 | 100 | SNPs | Informative for k=3 populations (European, Native American, African) | **European:** 42 European Americans  **Native American:** 15 Mayas, 15 Nahua  **African:** 37 West Africans |
| Galanter, *et al.*, 2012 | 446 | SNPs | Informative for k=3 populations (European, Native American, African) | **European:** 56 CEU, 55 TSI, 619 Spanish  **Native American:** 25 Aymara, 24 Quechua, 14 Nahua, 25 Mayas, 22 Tepehuanos, 21 Zapotecas  **African:** 53 Yorubas, 50 Kenya |
