# Supplementary material for: A panel of 32 AIMs suitable for population stratification correction and global ancestry estimation in Mexican mestizos
Source: BMC Genet. 2019 Jan 8;20:5. doi: 10.1186/s12863-018-0707-7 (PMC6323778; doi:10.1186/s12863-018-0707-7)
Supplement: Supplementary file 3 — Table S2. Panel of 32 AIMs proposed in this study and allele frequencies. (DOCX 16 kb) [file 12863_2018_707_MOESM3_ESM.docx]

| **Additional Table 2. Panel of 32 AIMs proposed in this study and allele frequencies.** | | | | | | | | |
| --- | --- | --- | --- | --- | --- | --- | --- | --- |
|  |  |  |  |  | **1000G** | **MGDP** | **SIGMA** | **Delta** |
| **SNP** | **CHR** | **POSITION** | **A1** | **A2** | **CEU** | **NAT** | **MEX** | **EUR/>95% NAT** |
| rs3843249 | 1 | 1373373 | G | A | 0.868 | 0.026 | 0.320 | 0.842 |
| rs9659240 | 1 | 204761062 | T | C | 0.916 | 0.026 | 0.336 | 0.889 |
| rs3755095 | 2 | 80097279 | T | C | 0.926 | 0.079 | 0.359 | 0.847 |
| rs3827760 | 2 | 109513601 | A | G | 1.0 | 0.066 | 0.408 | 0.934 |
| rs10510511 | 3 | 21260370 | G | T | 0.979 | 0.118 | 0.435 | 0.861 |
| rs12495357 | 3 | 64514008 | G | A | 0.990 | 0.105 | 0.405 | 0.884 |
| rs67929453 | 3 | 139109825 | A | G | 0.868 | 0.079 | 0.381 | 0.789 |
| rs10016699 | 4 | 61822587 | C | T | 0.958 | 0.158 | 0.409 | 0.800 |
| rs4833808 | 4 | 122992499 | T | C | 0.842 | 0.079 | 0.367 | 0.763 |
| rs35407 | 5 | 33946571 | G | A | 0.984 | 0.092 | 0.351 | 0.892 |
| rs12521018 | 5 | 167266430 | C | T | 0.842 | 0.145 | 0.421 | 0.697 |
| rs12529753 | 6 | 56191582 | T | C | 0.932 | 0.081 | 0.376 | 0.851 |
| rs9406333 | 6 | 169718094 | T | G | 0.926 | 0.039 | 0.404 | 0.887 |
| rs1858892 | 7 | 113282616 | A | G | 0.800 | 0.079 | 0.310 | 0.721 |
| rs61097563 | 8 | 72429909 | C | T | 0.053 | 0.829 | 0.509 | -0.776 |
| rs12549875 | 8 | 91207269 | T | C | 0.953 | 0.211 | 0.436 | 0.742 |
| rs10116041 | 9 | 18263296 | A | G | 0.884 | 0.039 | 0.382 | 0.845 |
| rs57432666 | 9 | 84237469 | G | A | 0.905 | 0.092 | 0.389 | 0.813 |
| rs734241 | 10 | 115239602 | G | A | 0.911 | 0.066 | 0.398 | 0.845 |
| rs1533224 | 11 | 80584598 | A | C | 0.758 | 0.105 | 0.350 | 0.653 |
| rs11612312 | 12 | 52349088 | T | C | 0.795 | 0.039 | 0.325 | 0.755 |
| rs1409264 | 13 | 93425688 | C | A | 0.790 | 0.079 | 0.345 | 0.711 |
| rs1243370 | 14 | 21674214 | T | C | 0.932 | 0.145 | 0.409 | 0.787 |
| rs4904274 | 14 | 86268151 | A | G | 0.0 | 0.750 | 0.470 | -0.750 |
| rs1426654 | 15 | 48426484 | A | G | 1.0 | 0.066 | 0.405 | 0.934 |
| rs10794640 | 16 | 789618 | G | A | 0.842 | 0.054 | 0.344 | 0.788 |
| rs59021505 | 16 | 10994136 | T | C | 0.995 | 0.066 | 0.432 | 0.929 |
| rs11657785 | 17 | 4400392 | T | C | 0.863 | 0.066 | 0.329 | 0.797 |
| rs7259453 | 19 | 46766979 | T | C | 0.811 | 0.158 | 0.374 | 0.653 |
| rs1418029 | 20 | 2060151 | G | A | 0.937 | 0.342 | 0.452 | 0.595 |
| rs9975044 | 21 | 29878029 | C | T | 0.911 | 0.079 | 0.374 | 0.832 |
| rs743832 | 22 | 42095464 | T | C | 0.037 | 0.645 | 0.505 | -0.608 |
| Allele frequencies of the panel of 32 AIMs were obtained from 1000 Genomes Project (CEU: Europeans), from MGDP (NAT: Native individuals from Mexico), as well as from SIGMA Consortium (MEX: Mexican mestizos). Positions correspond to build 37. | | | | | | | | |
