# Supplementary material for: A panel of 32 AIMs suitable for population stratification correction and global ancestry estimation in Mexican mestizos
Source: BMC Genet. 2019 Jan 8;20:5. doi: 10.1186/s12863-018-0707-7 (PMC6323778; doi:10.1186/s12863-018-0707-7)
Supplement: Supplementary file 4 — Table S3. Commercial genotyping arrays where our subset of 32 AIMs is available. (DOCX 13 kb) [file 12863_2018_707_MOESM4_ESM.docx]

| **Additional Table 3. Commercial genotyping arrays where our subset of 32 AIMs is available.** | |
| --- | --- |
| **SNP** | **Arrays** |
| rs3843249 | O24,O28 |
| rs9659240 | OQ,AAH,O24,O28 |
| rs3755095 | I3,I5,I6,I6Q,IM,IMD,IC,ICQ,OQ,IWQ,OE,O24,O28,O54,O5E,OEE |
| rs3827760 | AG,I1,IM,IMD,CYT,OQ,AxM,OE,O24,AAE |
| rs10510511 | AH,AN,A5,A6,I2,I5,I6,I6Q,IM,IMD,CYT,OQ,AxM,IWQ,OE,O24,O28,O54,O5E,OEE,AAH |
| rs12495357 | O24,O28,O54 |
| rs67929453 | O24,O28 |
| rs10016699 | A6,O24,O28 |
| rs4833808 | IM,IMD,OQ,AxM,OE,O24,O28,O54,O5E,OEE |
| rs35407 | A6,I1,IM,IMD,OQ,AxM,OE,O24,O28,O54,O5E,OEE |
| rs12521018 | O24,O28 |
| rs12529753 | O24,O28 |
| rs9406333 | I3,I5,I6,I6Q,IM,IMD,IC,ICQ,CYT,OQ,IWQ,OE,O24,O28,O54,O5E,OEE |
| rs1858892 | A6,IM,IMD,AxM,O24,O28 |
| rs61097563 | O24,O28,O54 |
| rs12549875 | O24,O28 |
| rs10116041 | A6,AxM,O24,O28 |
| rs57432666 | O24,O28,O54 |
| rs734241 | I2,I5,I6,I6Q,IM,IMD,CYT,OQ,IWQ,OE,O24,O28,O54,O5E,OEE |
| rs1533224 | AX,I3,I5,I6,I6Q,IM,IMD,IC,ICQ,OQ,AxM,IWQ,OE,O24,O28,O54,O5E,OEE,A10 |
| rs11612312 | I2,I5,I6,I6Q,IM,IMD,OQ,IWQ,OE,O24,O28,O54,O5E,OEE |
| rs1409264 | AN,A5,A6,OQ,OE,E1,E11,O24,O28,O54,O5E,OEE,AAE |
| rs1243370 | I2,I5,I6,I6Q,IM,IMD,CYT,OQ,AxM,IWQ,OE,O24,O28,O54,O5E,OEE |
| rs4904274 | AS,A5,A6,I2,I5,I6,I6Q,IM,IMD,OQ,IWQ,OE,O24,O28,O54,O5E,OEE,AAH |
| rs1426654 | AG,I1,I6Q,IM,IMD,CYT,OQ,AxM,IWQ,OE,O24,AAE |
| rs10794640 | O24,O28,O54 |
| rs59021505 | O24,O28,O54 |
| rs11657785 | O24,O28,O54,O5E |
| rs7259453 | I3,I5,I6,I6Q,IM,IMD,IC,ICQ,OQ,IWQ,OE,O24,O28,O54,O5E,OEE |
| rs1418029 | A6,AxM,AAH,O24,O28 |
| rs9975044 | O24,O28,O54 |
| rs743832 | IM,IMD,CYT,OQ,AxM,OE,O24,O28,O54,O5E,OEE |
| Illumina Human-1 (I1), Illumina HumanHap240S (I2), Illumina HumanHap300 (I3), Illumina Human370CNV single (IC), Illumina Human370CNV quad (ICQ), Illumina HumanHap550 (I5), Illumina HumanHap650 (I6), Illumina HumanHap610 quad (I6Q), Illumina Human1M single (IM), Illumina Human1M dual (IMD), Illumina CARe iSelect (IBC), Illumina Cyto12 (CYT), Illumina OmniQuad (OQ), Cardio-Metabochip (CM), Illumina 660W-Quad (IWQ), Illumina OmniExpress (OE), Human Exome 12v1 (E1), Human Exome 12v1.1 (E11), OmniChip 2.5M 4 sam (O24), OmniChip 2.5M 8 sam (O28), OmniChip 5M 4 sam (O54), Omni 5 Exome (O5E), Omni Express Exome (OEE), Immuno BeadChip A (ICA), Immuno BeadChip B (ICB), Affymetrix 50K Mapping XbaI (AX), Affymetrix 50K Mapping HindIII (AH), Affymetrix 50K Human Gene Focused chip (AG), Affymetrix 250K Mapping NspI (AN), Affymetrix 250K Mapping StyI (AS), Affymetrix 5.0 (A5), Affymetrix 6.0 (A6), Affymetrix Axiom GW_Hu_SNP (AxM), Affymetrix DMET plus (AD), Affymetrix 10K Mapping Array bld2 (A10), Affymetrix Axiom Exome Plus Array (AAE), Affymetrix Axiom GW Hu Origins 1 (AAH). Information obtained from the SNP Annotation and Proxy Search v2.2 web page (http://www.broadinstitute.org/mpg/snap/ldsearch.php). | |
